# A New Method for Postural Misalignment of a 6-Year-Old Girl With Cerebral Palsy: A Case Report

**DOI:** 10.1016/j.arrct.2021.100116

**Published:** 2021-02-23

**Authors:** Ying Hou, Huitian Zheng, Jinping Li, Shujia Wang, Dongmei Zhang, Tong Tang, Mindan Xu, Hong Zhou

**Affiliations:** a Department of Rehabilitation Medicine, Affiliated Suzhou Hospital of Nanjing Medical University, Suzhou; b Neurological Rehabilitation Department, Zhangjiagang Gangcheng Rehabilitation Hospital, Zhangjiagang; c Children Rehabilitation Department, Kunshan Rehabilitation Hospital, Kunshan, China

**Keywords:** Cerebral palsy, Exercise movement techniques, Posture, Rehabilitation, *List of abbreviations:* CP, cerebral palsy, CST, Constraint Standing Training, GMFCS, Gross Motor Function Classification System, GMFM-88, Gross Motor Function Measure-88, IC, initial contact, KFA, knee flexion angle, MMT, manual muscle testing.

## Abstract

**Objective:**

To demonstrate the effects of a newly designed postural alignment relearning system on postural control dysfunction in a typical patient with cerebral palsy (CP).

**Design:**

Evaluation before and after 8 weeks of Constraint Standing Training 3-dimensional postural alignment relearning system.

**Setting:**

Department of Rehabilitation Medicine.

**Participant:**

A 6-year-old girl with CP and postural misalignment on Gross Motor Function Classification System level I.

**Interventions:**

Constraint Standing Training for 8 weeks to correct postural misalignment.

**Main Outcome Measures:**

Parameters of lateral plain radiographs in static standing, posturography measurements in standing and walking, motor ability (Gross Motor Function Measure-88 [GMFM-88] scores, manual muscle testing [MMT] scores, muscle architecture), and gait kinematic parameters (40 3-dimensional parameters of arms, trunk, waist, and lower limbs).

**Results:**

Knee hyperextension angle in static standing; peaks of knee flexion angle (KFA) when walking, hip flexion angle and ankle flexion angle in dynamic standing; and the KFA at initial contact in gait cycle all decreased significantly (*P*<.01). Scores of GMFM-88 sections D and E and MMT of 5 core stability muscles improved (*P*<.01). The velocities and range of motion of the arms, the 3-dimensinoal range of motion of the trunk and waist, and most of the parameters of the lower limbs showed statistically significant change (*P<*.01). Bilateral muscle thickness did not change significantly after the treatment (*P=*.738 left, *P*=.978 right), but the gluteus maximus morphology was changed: the muscle fibers became rounder, the interfiber space decreased, and the border lines of the muscle fibers got clearer.

**Conclusions:**

Postural alignment, motor ability, and gait may be homologous external manifestations of more fundamental core abilities, referring to correct standing posture cognition, muscle activation, and postural unconsciousness. Constraint Standing Training 3-dimensional postural alignment relearning system aimed to improve the static and dynamic standing control ability, may fix postural misalignment and improve motor ability and flexed-knee gait. Future work should use Constraint Standing Training with patients with different kinds of misalignment, choose sensitive indicators, observe the duration of each step, and reveal the mechanism causes postural misalignment.

Postural control dysfunction in children with cerebral palsy (CP), especially in Gross Motor Function Classification System (GMFCS) level I-III, is common.^1^, ^2^, ^3^ Patients with postural control dysfunction are unable to coordinate the activation of postural muscles in the correct sequence,^4^^,^^5^ and the developed anticipatory posture can be variable and ineffective.^6^ At this time, there is no 1 standard therapy recommended because it is not clear what element causes this dysfunction.^7^

We focused on postural misalignment as the starting point for research because the posture orientation is 1 of the 2 elements involved with postural control.^8^ Postural misalignments associated with CP, such as genu recurvatum, flexed-knee, pelvis tilt, changes of the spinal curvature, and increased femoral anteversion,^9^, ^10^, ^11^ recognized clinically as a complex multijoint, multiplanar postural orientation disorder,^8^ may result in muscle weakness, joint pain, and joint contracture.^12^^,^^13^ Multilevel surgeries aimed to cure joint contracture are used in clinical practice, but their effects on postural control were controversial.^14^^,^^15^ Previous reports have not illuminated the pathogenesis of postural misalignment in patients with CP nor its relationship with postural control dysfunction.^16^^,^^17^ To date, whether the postural misalignment can be prevented or cured and whether and how prevention or correction improve postural control is not clear.^7^

Our team has been working on and refining a postural alignment relearning system since 2013, with the goal to change the postural misalignment of patients with postural control dysfunction. We now have a 5-step protocol titled Constraint Standing Training (CST) 3-dimensional postural alignment relearning system. Results from the previous protocol on pelvic anterior tilt and gait speed in children with CP have been published.^18^ In this article, we present the updated protocol for the first time and share the initial results of Constraint Standing Training on a 6-year-old girl with GMFCS level I CP. The goal of our research is to reveal the elements of postural misalignment and understand the correlation between postural alignment correction and postural control in children with CP.

## Methods

This was a phase 1 clinical study of the 3-dimensional postural alignment relearning system used with children with CP. This report was limited to 1 participant because of the limitation that the patient could not accept any other therapy during the trial, and most parents preferred to choose integrated physical therapy. The participant's guardian provided written informed consent as approved by our institutional review board. The trial was registered with the Chinese Clinical Trial Registry (no. ChiCTR1900022573) before implementation.

### Participant

The participant was a 6-year-old girl with GMFCS level I CP. She has speech delay, visual impairment, dysarthria, genu recurvatum, and flexed-knee gait. Previous rehabilitation over 4 years included language and speech therapies as well as visual and physical therapy, such as strength training, gait training, vibration, and balance training. With these therapies, her vision and linguistic function improved, but her motor dysfunction did not improve as significantly. Therefore, the parents’ therapeutic goal for participation in Constraint Standing Training was to improve the child's posture in standing and walking and to enable independent navigation of stairs. Initially her Gross Motor Function Measure-88 (GMFM-88) sections D and E scores were 89.7% and 91.6%, respectively. On examination, the participant demonstrated hyperkyphosis, lumbar hypolordosis, knee hyperextension, and flexed-knee gait but did not have obvious disorder in reflexes, range of motion, sensation, cognition, or autonomic nerve function. There was no family history of any genetic disease.

### Procedures

The plan was for the girl to participate in the revised Constraint Standing Training, for 60 sessions (30min/d, 5d/wk for 12wk). She did not receive any other motor or gait treatment during the training. A full battery of standardized tests, including undressing, ultrasonic testing, watching videos for postural imitation, assessments of postural alignment, motor ability, gait, and dressing were performed before and after the treatment by a physician and 2 rehabilitation therapists. Portions of tests of motor ability were performed every 2 weeks during the treatment.

This study was approved by Ethics Committee of The Affiliated Suzhou Hospital of Nanjing Medical University.

### Standardized testing

Measures of posture morphology, GMFM-88 and manual muscle testing (MMT), muscle architecture via ultrasonography, and gait assessment were evaluated by a skilled physician and 2 rehabilitation therapists. The standards in the measures, including the location of the camera; the instructions given in standing, walking, and other motor assessments; the intensity of pressure of ultrasonic probe on skin; and the assessment environment, were all established before the trial.

### Protocol of Constraint Standing Training

**Fig. 1:**
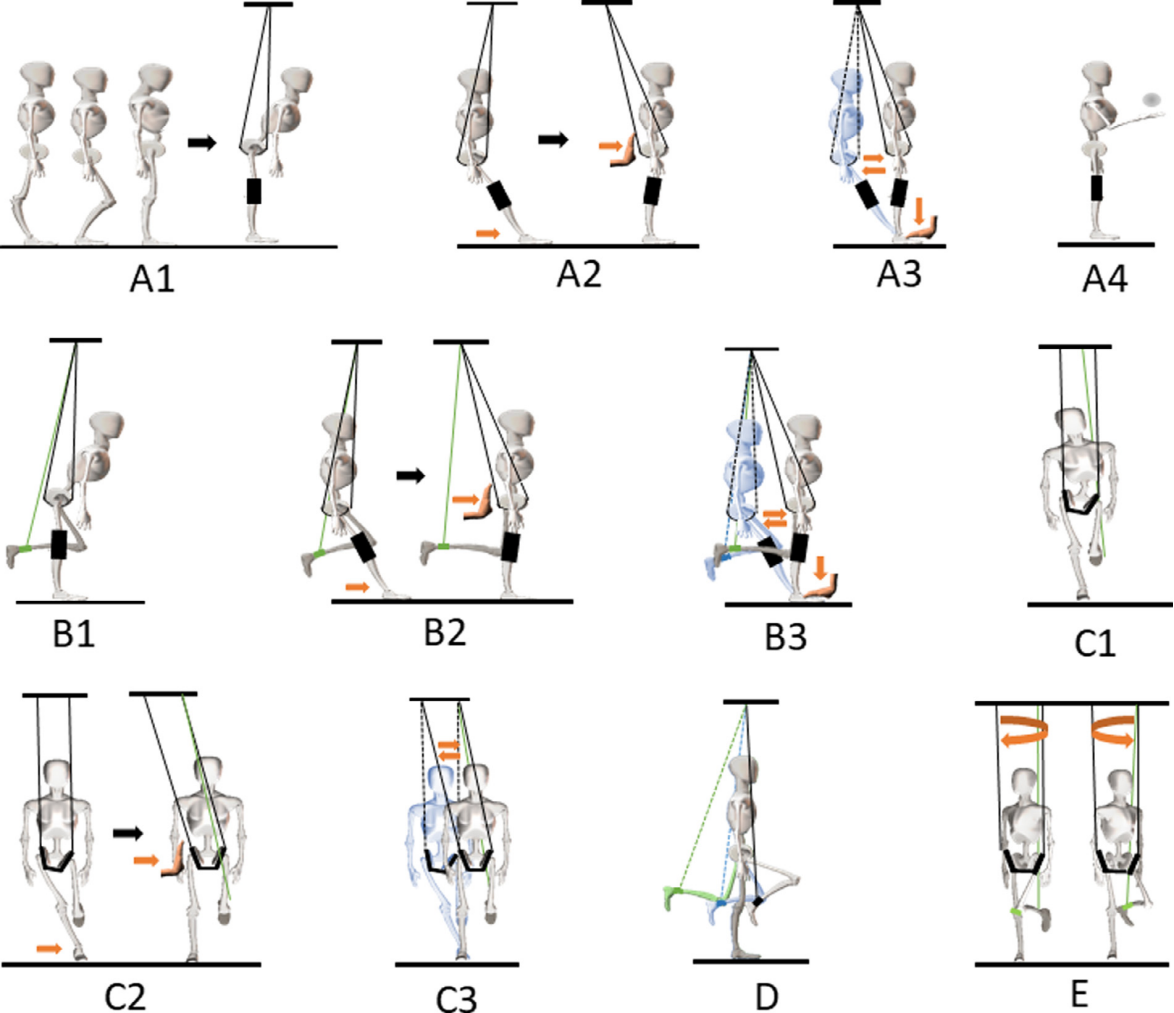
Protocol for the 5 steps of CST. Abbreviations: CST, Constraint Standing Training.

Weight support equipment, Weight Support Gait Trainer,^a^ was used to complete 5 steps of Constraint Standing Training as follows (fig 1):

#### Step A: sagittal alignment correction in both legs standing

A1: misalignment conversion. The patient, with 1 or more misalignments of lumbar curvature, pelvic tilt, couch standing, or genu recurvatum and without severe joint contracture or acute joint and bone injury, stands wearing a pair of knee splints and a harness hanging on the weight support equipment. Once the knees fix straight, the postural misalignments mentioned above are converted to the hip flexion posture.

A2: feet position adjustment. Move the feet forward as far as possible until the extended hips are constrained and the legs are vertical.

A3: posture relearning. Fix the feet and help the patient to swing hips back and forth. Induce the patient to stand with hips extended as long as possible. During this step, the patient starts to relearn the proper postural alignment in sagittal plane, with neutral pelvis, straight legs, and vertical tibia shaft anatomic axis. When hip extension can be maintained easily, unfix the knee splints and gradually increase knee extension duration and strength in hips extension position.

A4: unconscious posture control. Add 1 or more secondary arm motor tasks when the patient is standing, such as waving, throwing balls, or giving pushes. Keep proper postural alignment during the process.

#### Step B: sagittal alignment correction in single leg standing

B1-B3: the same as A1-A3 but with 1 leg standing and the other hanging on an elastic strap. During step B3, help the patient to avoid unstable swaying from side to side.

#### Step C: coronal alignment correction in single leg standing

C1: misalignment conversion. The patient stands on a single foot with the other hanging on an elastic strap, wearing a harness hanging on the weight support equipment. After steps A and B, the patient can stand staight in sagittal plane without knee splints. But it is still difficult for the patient to stand straight on a single foot without help in coronal plane. It can be observed that the patient's spine bends to the weight-bearing side, and the pelvis tilts to the opposite side in single leg standing.

C2: foot position adjustment. Move the standing foot inward as far as possible until the abducted hips are constrained and the standing leg is vertical.

C3: posture relearning. Fix the standing foot and help the patient to swing the hips side to side. Induce the patient to stand with the hip abducted as long as possible. During this step, the patient starts to relearn the proper prostural alignment in coronal plane with neutral pelvis and erect spine.

#### Step D: dynamic stability of single leg standing

The patient stands on a single foot, with the other hanging and swinging back and forth. Keep the standing leg as stable as possible.

#### Step E: Horizontal dynamic stability

The patient stands on a single foot while the pelvis and trunk rotate horizontally. Keep the standing leg as stable as possible and avoid knee rotation.

### Measures

#### Lateral plain radiographs in static standing^19^

Lateral plain radiographs of the whole body in static standing were taken before and after the treatment with the patient standing as straight as possible. Thoracic kyphosis, thoracolumbar kyphosis, lumbar lordosis, pelvic incidence, sacral slope, pelvic tilt, knee hyperextension angle, and plantigrade angle were measured in computer-aided design software (AutoCAD).^b^

### Posturography measurements

Posturography and videos were captured with a digital single lens reflex camera (Canon EOS 6D).^c^ The camera was fixed on top of a tripod 1-m high at a 1.5-m distance from the background. The patient was asked to watch a standard teaching video in which a 6-year-old boy stands straight and waves his arms. She was then asked to perform as follows: (1) wave arms overhead when standing, for 3 attempts, while the peaks of knee flexion angle (KFA), hip flexion angle, and ankle flexion angle were measured in AutoCAD software; (2) walk back and forth in a 20-m trail at a self-selected comfortable walking speed, while the KFA at initial contact (IC) of gait cycle was measured in AutoCAD software; and (3) stand on a single straight leg and then with both legs straight, as long as possible, for 3 attempts. The longest durations of proper standing posture on single and then both legs (without pelvic tilt, knee hyperextension, or flexion) of 3 tests were recorded as the results.

### Gross Motor Function Measure-88^20^

The patient functions at GMFCS level I. She had ability in lying, rolling, sitting, crawling, and kneeling. Section D contains 13 items and refers to standing function. The scores range from 0-39. Section E contains 24 items and refers to walking, running, and jumping functions. The scores range from 0-72. The results are presented as percentages.

### Manual muscle testing^21^

MMT scores 0-5 reflect the strength of 6 muscles: abdominis, gluteus maximus, gluteus medius, quadriceps femoris, hamstring muscles, and triceps surae.

### Muscle architecture measurement

We measured and recorded the architecture of 20 lower limb muscles via a high-frequency ultrasonography system (X-Porte TTC).^d^

### Gait assessment with wearable inertial sensors^22^

Opal sensors (Mobility Lab v2)^e^ were secured on the participant at the following locations: (1) on the wrist, worn like 2 watches; (2) centered on a flat surface at the third rib level; (3) centered on the middle of the low back at the base of the spine; and (4) centered on the dorsum of the feet. The participant was commanded to walk barefoot, back and forth, on a 20-m trail at a self-selected comfortable walking speed for 2 minutes. The device can detect 40 parameters during a walk test, including 4 arms parameters, velocities and range of motion of the arms, 6 axial parameters, the 3-dimenstional ranges of motion of the trunk and lumbar, 20 parameters of motion of the lower limbs, bilateral cadence, elevation at midswing, circumduction, gait speed, gait cycle duration, step duration, stride length, angles of foot strike, toe out and toe off, 10 ratios in gait cycle, bilateral double support, terminal double support, single limb support, stance, and swing.

### Analyses

Parameters before and after Constraint Standing Training were reported. Nonparametric statistics were described. Smallest real difference and minimum clinically important difference were used to interpret scores. A paired *t* test was used to compare the numeric data. Calculations were performed using SPSS software.^f^

## Results

The patient participated in Constraint Standing Training for 30 sessions, 30 min/d, 3-4 days a week for 8 weeks. She only completed steps A and B of Constraint Standing Training because she needed to go to primary school after the 8 weeks of therapy and did not have time to finish Constraint Standing Training. In addition, her parents were satisfied with the improvement at that time; after 4 weeks, she could walk up and down the stairs independently, and after 8 weeks, her motor skill improved to be more flexible and stable. The treatment process was going well, and the patient had not complained about having pain or difficulty.

### Lateral plain radiographs in static standing

**Fig. 2:**
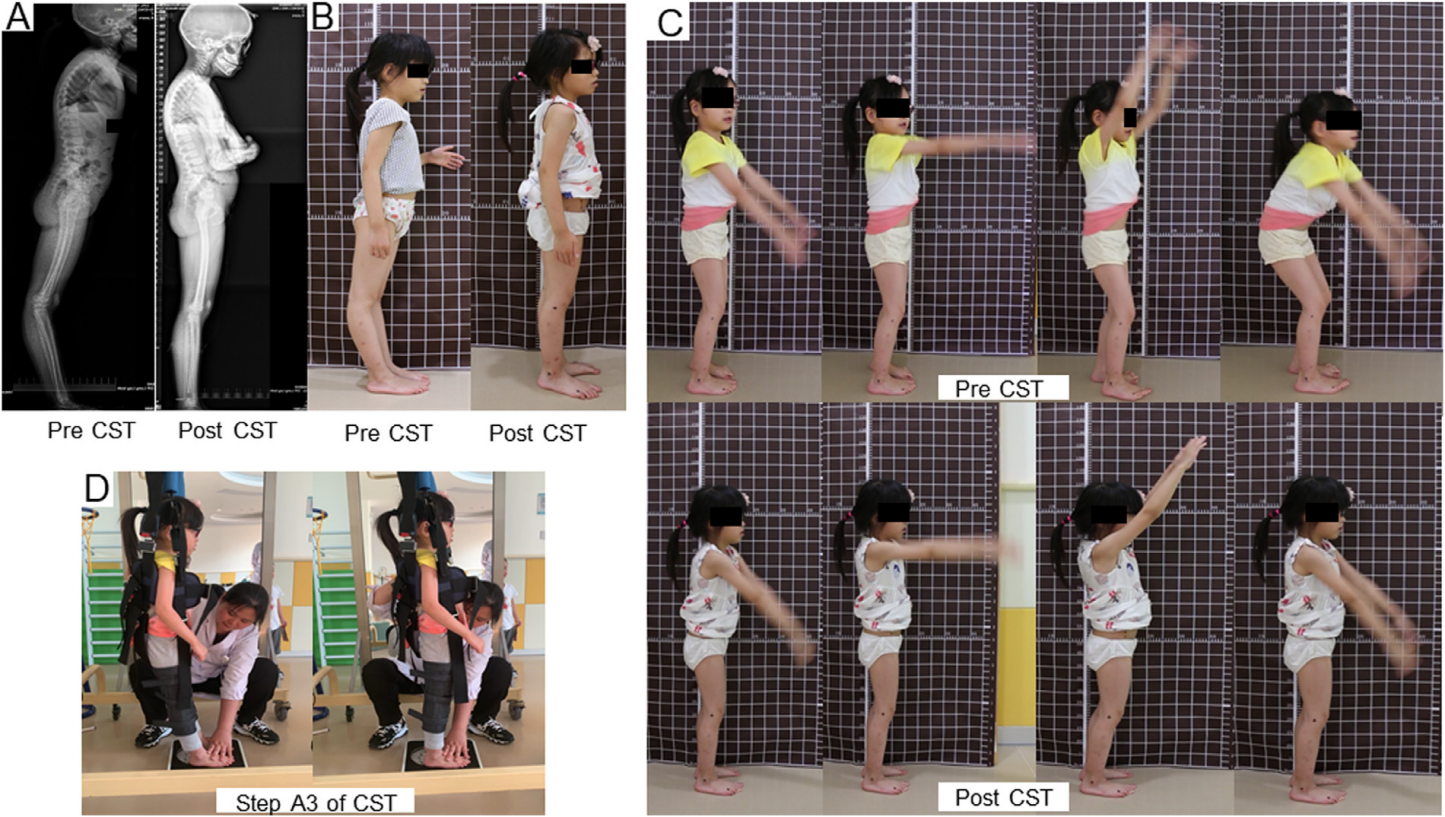
Static and dynamic postural alignment before and after CST. **(A)** Lateral plain radiographs of the whole body showed that the patient had hyperkyphosis, lumbar hypolordosis, posterior pelvic tilt, and knee hyperextension. After the treatment, knee hyperextension recovered. **(B)** The static standing posturographies from the right-side view. **(C)** Dynamic stability alignment of lower limbs with arms waving overhead before and after CST showing that the patient could keep knee straight unconsciously after CST. **(D)** Step A3 of CST. The physical therapist fixes the patient's feet and helps the patient swing her pelvis in sagittal plane and then induces her to maintain the position.

Abbreviations: CST,Constraint Standing Training; Pre-CST, before Constraint Standing Training. Post-CST, after Constraint Standing Training.

Angles of knee hyperextension angle and plantigrade angle improved from 27.8°and 102.1° to 1.8° and 89.0°, respectively. Angles of the spine—thoracic kyphosis, thoracolumbar kyphosis, lumbar lordosis, pelvic incidence, and sacral slope—did not change significantly (table 1, fig 2A,B).

**Table 1:** Postural alignment and motor ability changes before and after CST

Items	Pre-CST	4 Wk	6 Wk	Post-CST	Changes	Description
Radiographic parameters in standing of sagittal plane (degree)						
TK	47.8	-	-	46.6	−1.2	Hyperkyphosis was not changed.
TL	15.0	-	-	18.8	3.8	
LL	−27.6	-	-	−26.4	1.2	Lumbar hypolordosis was not changed.
PI	31.7	-	-	28.0	−3.7	Posterior pelvic tilt was not changed.
SS	23.1	-	-	10.7	−12.4	
PT	8.6	-	-	17.3	8.7	
KHA	27.8	-	-	1.8	−27.6	Knee hyperextension in standing recovered.
PGA	102.1	-	-	89.0	−13.1	Tibias became more vertical.
Peak flexion angles when waving arms (degree)						
HFA	65.0	-	-	14.7	−50.3	Dynamic stability of postural alignment was acquired.
KFA	54.0	-	-	15.0	-−9.0	
AFA	9.4	-	-	9.3	−0.1	
KFA at IC when walking (degree)						
Left KFA	35.0±6.83	-	-	13.8±4.4		Flexed-knee gait improved.
Right KFA	36.4±6.4	-	-	10.5±3.1		
GMFCS	I	I	I	I	-	
GMFM-88 scores (%)						
D section	89.7	89.7	94.87	100	10.3	Improvements found in the following items: 57, 58, 60, 61.
E section	91.6	68.0	70.0	98.6	7.0	Improvements found in the following items: 74, 78-83, 86-88.
Strength: MMT						
MMT: abdominis	4	4	5	5	1	Strengths increased after 6 weeks of the treatment.
MMT: gluteus maximus	4	4	5	5	1	
MMT: gluteus medius	4	4	4	5	1	
MMT: quadriceps femoris	4	4	5	5	1	
MMT: hamstring muscles	4	4	5	5	1	
MMT: triceps surae	4	4	4	4	0	
Postural Durations						
Duration: standing on both feet (min)	0	5	>30	>60	>60	She could stand in proper postural alignment for 5 s on the first day of treatment.
Duration: standing on left foot (s)	3	8	34	55	52	
Duration: standing on right foot (s)	3	9	38	63	60	

Abbreviations: AFA, ankle flexion angle; CST, Constraint Standing Training; HFA, knee flexion angle; KHA, knee hyperextension angle; LL, lumbar lordosis; PGA, plantigrade angle; PI, pelvic incidence; Pre-CST, before Constraint Standing Training. Post-CST, after Constraint Standing Training; PT, pelvic tilt; SS, sacral slope; TK, thoracic kyphosis; TL, thoracolumbar kyphosis.

### Posturography measurements

**Fig. 3:**
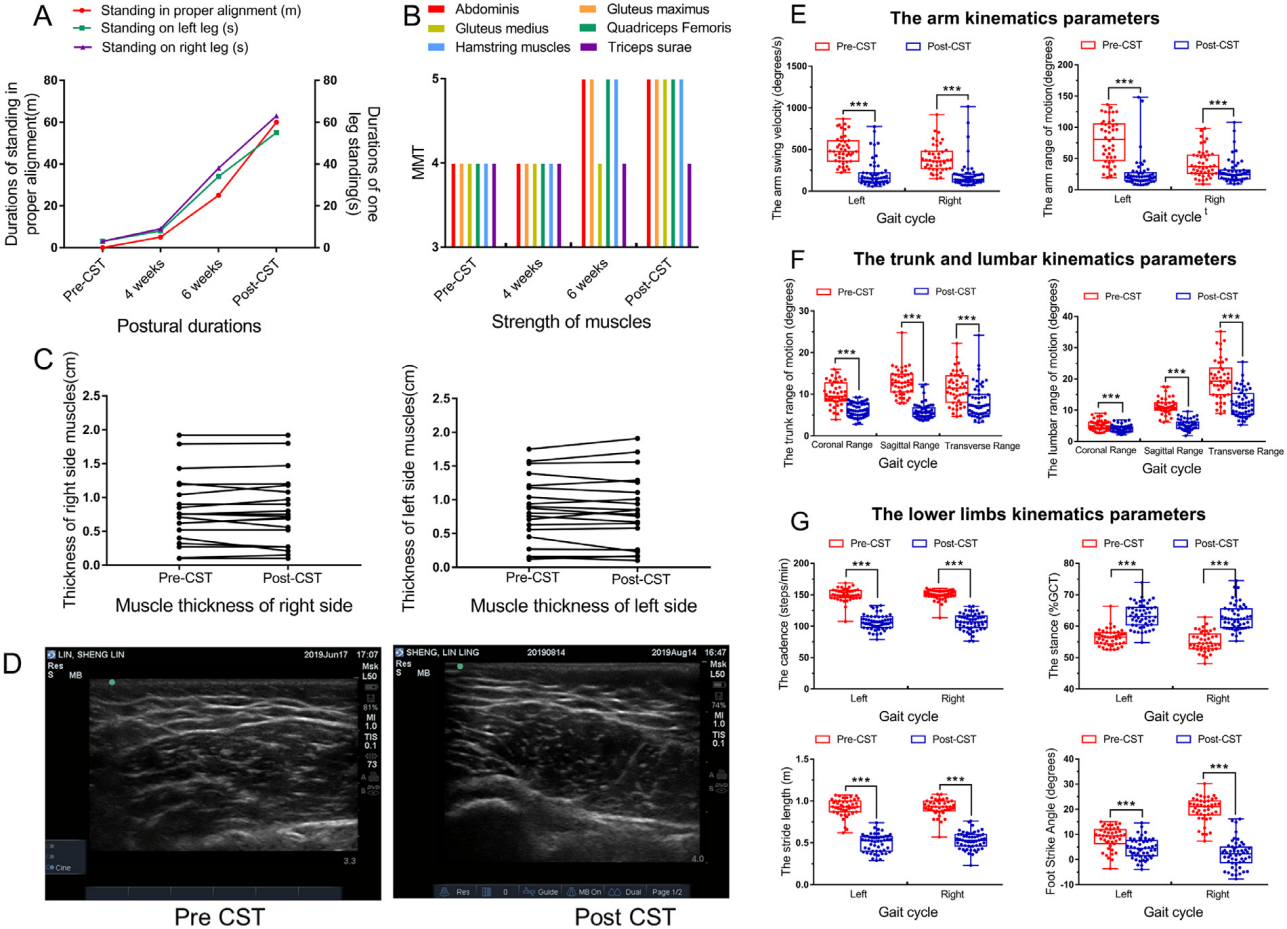
Motor ability and gait parameters of the patient before and after CST. **(A)** Postural durations began to increase in the first 4 weeks and increased faster in the latter 4 weeks. **(B)** Strength of muscles before and after CST shows that the strength of the global core muscles began to increase after 6 weeks of treatment. **(C)** Bilateral muscle thickness before and after CST shows that muscle thickness was not changed significantly, *P*=.738 (left), *P*=.978 (right). **(D)** Muscle ultrasonoscopies of the right gluteus maximus before and after CST. Muscle thickness was not changed, but the gluteus maximus morphology was changed: the muscle fibers became rounder, the interfiber space decreased, and the border lines of the muscle fibers got clearer. **(E)** Velocities and range of motion of the arms all decreased significantly (*P*<.01). **(F)** 3-dimensional range of motion of the lumbar and trunk all decreased significantly (*P*<.01). **(G)** Gait parameters of the lower limbs showed that the cadences decreased and the ratios of stance, stride length, and foot strike angle were changed significantly (*P*<0.01).

Abbreviations: CST, Constraint Standing Training; Pre-CST, before Constraint Standing Training. Post-CST, after Constraint Standing Training.*P<.01.

Peak hip flexion angle, KFA, and ankle flexion angle in dynamic standing and KFA at IC of gait cycle showed significant improvement (*P<*.01) (see table 1, fig 2C). On the first day of treatment with Constraint Standing Training the patient could keep proper postural alignment briefly in step A3 (see fig 1A, fig 2D). Durations of proper posture increased nonlinearly during the 8 weeks (see table 1, fig 3A).

### GMFM-88 scores

Scores of sections D and E were improved (see table 1). In section D, the improved abilities included the following items: lifts each foot with arms free for 10 seconds when standing and attains a standing position through half kneel without using arms when high-kneeling on each knee. In section E, scores were advanced in the following items: walks forward 10 consecutive steps on a straight line 2-cm wide, kicks ball with each foot, jumps 30-cm high with both feet simultaneously, jumps forward 30 cm with both feet simultaneously, hops on each foot 10 times within a 60-cm circle, jumps off with both feet simultaneously, and walks up and down 4 steps with alternating feet.

### MMT scores

MMT scores increased significantly for 5 of 6 muscles (*P=*.025) (see table 1, fig 3B).

### Muscle architecture via high-frequency ultrasonography

Bilateral muscle thickness via high-frequency ultrasonography did not change significantly after the treatment (*P=*.738, left; *P*=.978, right), but the gluteus maximus morphology was changed: the muscle fibers became rounder, the interfiber space decreased, and the border lines of the muscle fibers got clearer (see fig 3C,D).

### Gait assessment with wearable inertial sensors

Ten parameters increased significantly after Constraint Standing Training (*P<*.01): bilateral gait cycle duration, step duration, ratios of double support, stance, and terminal double support in gait cycle. A total of 26 parameters decreased significantly (*P<*.01): 4 parameters of the velocities and range of motion of the arms, 6 axial parameters of motion of the trunk and waist, 12 parameters of motion of the lower limbs, bilateral cadence, gait speed, stride length, angles of foot strike, toe off and toe out, 4 ratios of bilateral swing, and single limb support in gait cycle (see fig 3E-G).

## Discussion

Our objective was to evaluate the effects of Constraint Standing Training on postural alignment, motor ability, and gait in children with CP and try to find its underlying mechanisms.

### Generalizability

In recent years, many therapeutic methods were used to improve postural control ability in patients with CP, such as hippotherapy, muscle strength training, core stability training, standing frames, whole-body vibration, balance training, suit therapy, and surgical managements.^9^ These methods improve postural control in patients with CP in different ways and at different levels. However, we hypothesized that the ability to know proper standing alignment and how to keep it is more fundamental. The results showed that we have successfully changed the postural alignment of the lower limbs and improved motor ability and gait control.

### Postural alignment

The results showed that the joint angles of the patient's lower limbs became normal in static and dynamic standing. It indicates that Constraint Standing Training may fix the postural alignment of the lower limbs. The patient maintained proper postural alignment briefly even on the first day of the treatment. The postural relearning happened before an increase in muscle strength, which was observed in the latter 4 weeks. This indicates that the change in postural alignment may result initially from successful motor relearning (awareness of what the correct posture is and how to act it out) and muscle activation and then from increases of muscle strength acquired later. Eventually this relearned posture would be performed unconsciously when secondary motor tasks are added.^23^ In the present study, the alignment of the spine was not changed significantly; therefore, a long-term observation is needed.

### Motor ability

The results showed that GMFM-88 and MMT scores, postural durations, and morphology of the gluteus maximus improved (see table 1, fig 3A-D), but none of the muscles thickened. Increased muscle strength happened from the sixth week onward. This indicates that Constraint Standing Training may help children with CP to improve integrated motor ability, and postural alignment and integrated motor may not only be correlated^24^ but also homologous external manifestations of more fundamental core abilities.

### Gait

Children with knee hyperextension may walk with knee flexed or knee recurvatum depending on degrees of dorsiflexion of the ankles.^25^ This case had typical flexed-knee gait,^10^ with excessive dorsiflexion of ankles.^26^ The results showed that KFA at IC changed from 35.0±6.83 (left) and 36.4±6.4 (right) to 13.8±4.4 (left) and 10.5±3.1 (right); the foot strike angle changed from 8.6±4.39 (left) and 19.9±4.89 (right) to 4.52±4.01 (left) and 2.11±5.43 (right). This indicates that her flexed-knee gait improved significantly (*P*<.01). The patient did not have any kind of gait training during the treatment. Therefore, improvement of a single leg may mainly result from step B, 1 leg standing alignment correction of Constraint Standing Training. The KFA at IC may have changed more if she had been able to finish the last 3 steps.

As for the axial trunk and lumbar part, the 3-dimensional ranges of motion decreased. This indicates that her axial dynamic stability increased, which may result from advanced skeletal alignment.

In addition, the velocities and range of the arms decreased, which indicates that her upper limb motion, as a complementary strategy to maintain balance and posture control during walking,^27^ reduced and was freer. This may be indirect evidence of an increase in axial dynamic stability of her body.

Finally, the patient walked more slowly and had longer gait cycle duration and double support phase, which indicates that the gait pattern changed after treatment.

### Constraint Standing Training

Patients with CP and postural control dysfunction do not have the ability to acquire the proper postural alignment from imitating or muscle strength training.^28^ We designed a backward and sideways constraint condition for the pelvis to change alignment of the pelvis and feet in sagittal and coronal planes to induce the patient with misalignment to relearn the proper standing posture step by step. Unlike in other trainings, during Constraint Standing Training the goal of every step is accurate and limited to only 1 plane. So, it is easy and universal for patients with different misalignments. There are 5 steps in Constraint Standing Training (see fig 1). The patient moves to the next step when the current step is almost complete. Although the weight support system is used, it is not the weight support effect but the horizontal constraint which works.

### Study limitations

Findings were limited to a 6-year-old normally intelligent participant with CP and postural misalignment. The patient only had time to complete 2 steps of Constraint Standing Training. Some precision evaluation technologies for postural control ability assessment were not used, including a motion capture system, electroencephalogram, foot pressure, and electromyogram measurement equipment. The evaluators of the battery of tests were not blinded.

## Conclusions

Postural alignment, motor ability, and gait may be homologous external manifestations of more fundamental core abilities, including correct standing posture cognition, muscle activation, and postural unconsciousness. Constraint Standing Training 3-dimensional postural alignment relearning system aimed to improve the static and dynamic standing control ability, may fix postural misalignment and improve motor ability and flexed-knee gait. Future work should apply Constraint Standing Training with patients with different kinds of misalignments, choose sensitive indicators, observe the duration of each step, and reveal the mechanism causes postural misalignment.

## Suppliers

a. Weight Support Gait Trainer, BRE-DJZ; Ranbo.
b. AutoCAD; Autodesk.
c. Canon EOS 6D; Canon.
d. X-Porte TTC; FUJIFILM SonoSite.
e. Mobility Lab v2; APDM.
f. SPSS 24; IBM Corp.
